# Patient aggression experienced by professional nurses in acute psychiatric ward: South Africa

**DOI:** 10.4102/hsag.v29i0.2158

**Published:** 2024-02-28

**Authors:** Tshinanne G. Thandavhathu, Mary Maluleke, Ndidzulafhi S. Raliphaswa, Mphedziseni E. Rangwaneni, Thingahangwi C. Masutha, Ndivhaleni R. Lavhelani, Duppy Manyuma, Langanani C. Makhado, Takalani E. Thabathe, Lufuno M. Kharivhe, Vusiwana P. Letlalo, Mulatedzi P. Mulaudzi

**Affiliations:** 1 Department of Advanced Nursing Science, Faculty of Health Sciences, University of Venda, Thohoyandou, South Africa

**Keywords:** acute psychiatric ward, aggression, experience, psychiatric patient, professional nurse

## Abstract

**Background:**

Aggression of patients in hospital wards has become an endemic problem and professional nurses are particularly at high risk.

**Aim:**

This article presents the types of patient aggression experienced by professional nurses working in an acute psychiatric ward in Vhembe District, South Africa.

**Setting:**

Vhembe District, South Africa.

**Methods:**

A qualitative approach using exploratory, descriptive and contextual research design was used. Four hospitals were purposively selected and 10 professional nurses were conveniently sampled to participate in the study. Individual interviews were used to collect data, which were then analysed through Tesch Open Coding Method. Measures to ensure trustworthiness and ethical considerations were adhered to throughout the study.

**Results:**

This study shed some light on the professional nurses’ lived experiences regarding the types of aggression from patients in an acute psychiatric ward in Vhembe District. The types of aggression are physical aggression, destructive behaviour and verbal aggression.

**Conclusion:**

The findings show that the types of aggression to which professional nurses are exposed are overwhelming and the consequences are shocking. As a result, the health of professional nurses is compromised. Therefore, this study recommends further studies to determine the kind of support needed by professional nurses working in an acute psychiatric ward and to investigate the impact of aggression from patients in acute psychiatric ward with regard to the quality of care.

**Contribution:**

This article contributes to the body of knowledge regarding patients’ aggression in acute wards in Vhembe District, South Africa.

## Introduction

Patient aggression against professionals working in acute psychiatric inpatient wards is a global concern (Bryant-Genevie, Rao & Lopes-Cardozo 2021; National Nurses United 2021) and has become an integral part of healthcare professionals’ everyday lives (d’Ettorre & Pellicani 2017; Yeo et al. 2017). Nurses, as the frontline of most groups of healthcare professionals and in the longest direct contact with the inpatients during care, treatment and rehabilitation, are mostly exposed to the risk of patient aggression (Lepiešová et al. 2018; Michael 2021; Wiley 2021).

Studies focusing on the healthcare professionals caring for admitted patient in the psychiatric ward and the implications of patient aggression are well-documented. Research examining staff factors found that the incidence of violence was higher in wards where staff members were uncertain of their roles (Hauswirth 2021; Stuckey 2016) or where a larger proportion of shifts were worked by substitute nursing staff (Hauswirth 2021; Insecurity Insight 2021). A higher staff-to-patient ratio has been found to be related to increased violence (Hahn et al. 2020). Fewer incidents occurred when the staff-to-patient ratio approached 1:1 and when staff members set limits (Timori, Suryani and Sutini 2019). Increased violence has been found to be associated with a lower level of experience among nurses (Beck, Manderscheid & Buerhaus 2018), new employee status (Wiley 2021) or a lack of staff training in aggression control techniques (Michael 2021).

Studies investigating the significant implication of patient aggression on the professional performance of nurses found that it negatively affects the quality of care provided (Stevenson et al. 2015), contributes to increased levels of stress (Itzhaki et al. 2018), anger, fear or anxiety, post-traumatic stress disorder symptoms (Jeffery and Fuller 2016; Cheung, Lee and Yip 2017), guilt, self-blame and shame (Lawrence et al. 2020), reduces the mental and physical well-being (Maluleke and Van Wyk 2017), reduces the motivation of nurses (Dean, Butler and Cuddigan 2021), decreases job satisfaction (Joubert and Bhangan 2018), increases the intent to leave the organisation (Timor et al. 2019) and lowers the health-related quality of life (Moylan et al. 2016).

Furthermore, several research studies have been conducted on admitted patient aggression against healthcare professionals focusing, to a large extent, on staff factors (Mc Cann, Baird & Muir-Cochrane 2014), patient factor (Niu et al. 2019), environmental factors (Lozzino et al. 2015), the prevalence of patient aggression in the wards (Beck et al. 2018; Rosenthal, Byerly and Taylor 2018), what constitutes patient violence (Kelly et al. 2016) and experiences of nurses caring for an aggressive patient (Maluleke and Van Wyk 2017). This literature inadequately describes the types of patients’ aggression through the lens of psychiatric nurses working in inpatient settings who are at much greater risk of experiencing all types of patients’ aggression.

Therefore, this study investigated the psychiatric nurses’ personal experiences of the types of patient aggression within the context of their inpatient care, treatment and rehabilitation to provide a more holistic and rich description of the phenomenon of patient aggression which may inform future clinical practice. As a result, this article presents the types of patient aggression experienced by professional nurses working in Vhembe District, South Africa.

## Methods

### Setting

The study was conducted in Vhembe District in Limpopo Province, South Africa. Vhembe District has four local municipalities: Thulamela, Makhado, Collins Chabane and Musina. During this study, Vhembe District has six general hospitals that provide mental healthcare, treatment and rehabilitation services. Out of six, five of them have acute mental healthcare units, namely, Siloam, Tshilidzini, Donald Fraser, Elim and Malamulele hospitals. Musina hospital provides 72 h of observation as designated. The hospitals with acute wards admit both males and females. Patients are admitted with conditions such as schizophrenia, bipolar mood disorder – types 1 and 2, substance-induced psychosis, schizoaffective disorder and puerperal psychosis.

### Research approach

A qualitative approach with an explorative, descriptive and contextual design was used. This approach was suitable for this study as the aim was to determine the experiences of professional nurses regarding the types of patients’ aggression in acute mental healthcare units (Polit & Beck 2017). All sampled professional nurses were given an opportunity to narrate their lived experiences. This study was contextualised per the topic, the purpose, population and the setting, to accurately explore and describe the types of patients’ aggression as experienced by professional nurses as they care, treat and rehabilitate admitted patients in psychiatric wards, as described by Grove and Gray (2019) and Polit and Beck (2017).

### Population and sampling strategy

The study population consisted of professional nurses working in an acute psychiatric ward at general hospitals of Vhembe District in Limpopo Province. Professional nurses working in the selected hospitals were conveniently sampled. The convenient sampling technique is explained by Grove and Gray (2019) and Brink, Van der Walt and Van Rensburg (2018) as a selection of the most readily available persons as participants in a study. Therefore, in this study, the first author approached and selected professional nurses who were available at the time of arrival in the hospital and gave consent to participate. Then, these professional nurses met all the required criteria. Participants had to:

- be a qualified psychiatric nurse
- be working in an acute psychiatric unit.

Participants were recruited by the first author who visited the selected hospitals. A meeting was held with the potential participants where the study purpose was fully explained, and the information sheet was handed out to them.

### Data collection

Data collection was done in two phases, namely, preparation of the participants and preparation of the instrument.

#### Preparation of the participants

The potential participants who volunteered to be part of the study were telephonically contacted for an appointment. Before the interviews, professional nurses who volunteered to participate in the study were again informed that giving consent was voluntary and that they could withdraw at any time if they did not wish to continue. Information regarding the use of audio recorder was given to the participants for them to switch it off if they no longer wanted to continue with the study.

#### Preparation of the instrument

Two tape recorders with extra batteries were obtained to record simultaneously for backup in an event where one has a problem during interviews and transcribing process. The first author as the instrument (an interviewer) during data collection was competent with effective communication skills.

Individual in-depth interviews were used to collect data. Data were collected for 3 months (from June to August 2019). An open-ended question was asked as a point of departure, ‘As you are working with aggressive patients daily, what are the types of aggression you experience?’ The response was followed by probing until nothing new was said by the participant. The interviews were audio-recorded and transcribed verbatim. After each interview, an audiotape was played back to the participant to check if what was recorded is what the participant wished to say and to add when necessary. Interviews were conducted at the selected general hospital, pre-arranged room, and with professional nurses during lunchtimes. Each interview lasted for 30–40 min. By the 10th participant, no new information was coming forth. Therefore, the authors were satisfied that data saturation was achieved. No field notes were taken as the interview was recorded on the audio recorder.

### Data analysis

Data were analysed using the Tesch Open Coding Method (Creswell 2014). This involved reading transcripts twice to understand what professional nurses said. Data were then arranged into categories and subcategories using the actual words from professional nurses. Similar themes were allocated to a specific category that captured the same idea. These categories were compared and further allocated to overall categories. Finally, three themes emerged, which were realised about the types of patient aggression professional nurses experience while working in the acute psychiatric ward.

### Measure to ensure trustworthiness

The criteria to ensure trustworthiness as outlined by Polit and Beck (2017) were assured in this study, namely, credibility, transferability, dependability and conformability.

To ensure credibility, participants were visited four times; the first time was while giving the study information to all of them and recruiting; the second time was to find those who were willing to be part of the study. The third time was during data collection, and the fourth was after data collection for confirmation and member checking. An audio recording was played back to each participant after the interview.

Dependability was maintained by reporting and consulting with the research cohort group team and supervisors. Data were retained to ensure the accuracy and consistency of the information. It was also done by revisiting the participants to clarify what they said during the interviews. The researcher allows cross-checking of codes by other qualitative researcher experts to see whether the codes will be the same by meeting with the team members and discuss the themes. Transferability was ensured by a thick description of all the research processes clearly from the research design, study setting, target population, sampling procedure, and data collection methods so that even if another researcher repeated the same study, it would yield the same results. For conformability, information recorded during interviews was transcribed without any alteration. The researcher did not influence the responses and outcomes of the study.

### Ethical considerations

Ethical principles were observed throughout the study to protect the rights of the participants. Prior to the commencement of the study, ethical clearance was obtained from the Higher Degree Ethics Committee, Research and Publication Committee of University of Venda (reference number FHS/21/PDC/25/1301). Department of Health and four selected hospitals issued permission letters to allow the researcher to conduct the study.

### Informed consent

During the recruitment of participants, a full explanation of the study was narrated. The participants were informed that they were under no obligation to participate in the study and that they could withdraw at any point. Furthermore, an information pamphlet about the study and the contact numbers of supervisors was provided to all the participants in English in case they needed clarity to ensure voluntary participation. During the meeting with each participant for data collection, a full explanation of the study was provided again, and each participant voluntarily gave written informed consent.

Furthermore, permission to use the audio recorder for the interviews was obtained from participants. Deception of participants was avoided by explaining to the participants how the interview would be conducted and that there were no incentives to be given to the participants. Privacy was maintained by conducting the interview in a private room, and each participant was interviewed separately. For confidentiality, the participants’ real names were not used. Instead, each participant was given a pseudo-name.

## Findings

The results of this study are presented according to demographic description as well as discussion of themes.

### Demographic description

The study participants’ profile consists of gender and working experience, and each is presented separately below.

### Gender of participants

The study participants comprised of 10 participants with eight females and two males. Professional nurses in all participated hospitals are dominated by females than males. This is so based on the reality that the nursing profession was mainly female-dominant historically (Van der Heever, Van der Merwe & Crowley 2019). On the other hand, based on the author’s observation, rural-based hospitals have fewer male nurses than urban-based hospitals.

### Working experience

The working experiences of participated professional nurses range from 2 to 25 years. Three professional nurses (30%) have 2–5 years of experience, two professional nurses (20%) have 6–10 years of experience, and five of them (50%) have 10–25 years of experience. This indicates that most professional nurses do have great experience working in a mental healthcare unit.

## Themes

Three themes emerged from the analysed data, namely, physical aggression directed to nurses and other patients during delivery of nursing care, destructive behaviour aiming to damage of properties in the ward, and verbal aggression towards nurses during delivery of nursing care in the ward. These are presented and supported by quotes extracted from the participants’ examples mentioned about the types of aggression.

### Physical aggression directed to nurses and other patients during delivery of nursing care

The results revealed that participants have experienced physical aggression by patients towards nurses or other patients. Some patients use objects to fight nurses while others used their fists, teeth or legs. These resulted in victims sustaining injuries. The findings are supported by the following quotes:

‘[*B*]ehavior can be physical aggression whereby a patient fights nurses I once was being assaulted by a clap by an MHCU and I have a scar on my hand of being bitten by the patient and wrist twisted.’ (P3, PN, female, Exp 2yrs)

‘Patient can start being aggressive during serving of meals demanding more food than others when called to order he may throw a nurse with a bowl of hot soft porridge and sustained burns.’ (P10, PN, female, Exp 14yrs)

‘The patient will be fighting another patient. And when staff intervene, the patient then starts to fight nurses, throwing of the available nearby objects like a chair. I lost my middle finger where the patient throws a bin towards me which injured my hand and my finger was cut off.’ (P6, PN, male, Exp 7yrs)

Some participants reported that during the process of them rendering nursing care, they become victims of patients’ physical aggression, which results in them sustaining serious injuries. This was supported by the following quote:

‘The patient demanded to go home and when trying to explain to him that he is not discharged, he started to be aggressive and kicked me in the abdominal area. I went to the consult a doctor in causality via occupational health nurse and leave was granted for injury on duty.’ (P1, PN, female, Exp 9yrs)

‘I was handling an aggressive patient in the ward with other nurses and security officer when the patient overpowered me and sustain fracture on my left hand.’ (P9, PN, male, Exp 12yrs)

Another psychiatric patient picked up a wheelchair and threw it at a staff member. Other staff members had reported being scratched and molested by the patients. Similarly, Edward et al. (2014) report that common physical violence acts experienced by nurses from patients included being spat on, being hit, being pushed, scratched and kicked.

### Destructive behaviour aiming to damage of properties in the ward

The participants reported that patients become destructive and start to break windows and throw furniture. The following quotes supported this:

‘The patient was admitted with a history of destructive behaviour and assaulting people at home. He started breaking the windows in the ward, throwing chairs to nurses and other patients in the ward.’ (P5, PN, female, Exp 22yrs)

‘I was working night duty in the early morning hours when I heard a sound in the male toilets, and when I went there, I found a patient breaking the basin.’ (P8, PN, female, Exp 5yrs)

‘Sometimes patients kick furniture the chairs. If they portray this kind of behavior (297), I can see that something is wrong, and the patient will be angry. We try to find out what makes him angry.’ (P7, PN, male, Exp 24Yrs)

### Verbal aggression towards nurses during the delivery of nursing care in the ward

The findings of this study indicate that the participants have experienced verbal aggression from patients in the ward. Common types of verbal abuse against nurses include the use of vulgar words and the use of threatening words. The following quotes supported this:

‘When talking about the severity of aggression when trying to talk with him, the patient will start to insult you.’ (P2, PN, female, Exp 4yrs)

‘We admitted another patient and on admission, she looks calm. The following day she started to demand to go to work as she doesn’t deserve to be in the hospital. When trying to tell her that she will not go to work as she is admitted, she verbalizes that today one will die and there will be bloodshed.’ (P4, PN, female, Exp 18yrs)

The above quotes concur with the findings by Edward et al. (2014) which state that the types of verbal abuse against nurses include yelling, being cursed, being intimidated and being harassed with sexual language.

## Discussion

This study aimed to understand the psychiatric nurses’ personal experiences of the types of patient aggression within the context of caring, treating and rehabilitating aggressive patients. The study results revealed that the experiences of nurses could be placed in three types, namely, physical aggression, damage to property and verbal aggression, based on meaningful statements from participants.

Professional nurses working with psychiatric patients experience patient aggression almost every day that ranges from physical, verbal and destructive behaviour. Findings by Xing et al. (2015) indicate that nurses are more likely to experience physical violence because they are more likely to have direct physical contact with patients through their daily tasks, such as medication and drawing blood samples.

During the process of managing patient aggression, some get hurt and end up consulting. Furthermore, according to Edward et al. (2014), common physical violence experienced by nurses from patients included being spat on, hit, pushed, scratched and kicked.

Physical aggression is reportedly experienced not only by nurses but also by other patients. Findings by Maluleke and Van Wyk (2017) indicate that some patients became aggressive towards their fellow patients because they were not impressed with sharing resources within the ward. They feel jealous and threatened by new admissions in the ward or become aggressive when called to order.

The first type ‘physical aggression’ reports and describes professional nurses to be constantly exposed to unpredictable repeated physical violent behaviour from patients such as being grabbed, kicked, spit at, hit, slapped or strangled. These findings are in line with various authors including Maluleke and Van Wyk (2017); d’Ettorre and Pellicani (2017); Timori et al. (2019) and Stickings (2019) and contrast with findings from earlier studies including Niu et al. (2019) who found that nurses experienced sexual intimidation or harassment by patients. Hauswirth (2021) found that their physical aggression is directed towards self, that a patient picks and scratches their skin, hits themselves and pulls their hair, while Rosenthal et al. (2018) report that nurses experienced patients’ physical aggression towards an object that a patient slams the door, scatters clothing and makes a mess.

The present findings reveal that patient becomes angry due to failure to cope with ward rules and finding themselves in a close ward with a security officer and surrounded by strangers. Due to anger, they started to fight people around using whatever they come across using vulgar words. This was also supported by Xing et al. (2015) in their study findings that furniture in the ward, such as tables and chairs, should be placed in the proper position and be fixed to ensure that they cannot be moved freely.

Damage of property aggression is a different kind of aggressive behaviour that can consist, for example, of breaking crockery, throwing furniture through the window, breaking objects or setting a fire (Hoek and Braam 2017). The study findings mirror that in the study conducted by Gandré et al. (2017) and Zhou et al. (2015). The setting of fire by Varghese, Khakha and Chadda (2016) is not mentioned by participants in this study.

Above all, professional nurses indicated that after observing patient aggression, they intervene by trying to calm the patient, try to identify the cause of aggression and remove the patient from the environment that triggers aggression. On failure of nonpharmaceutical intervention, patients will be given treatment following protocols and doctor’s prescription, and they become calm after a few minutes. From the participants’ findings, if the patient does not get calm after giving treatment, the patient is taken to the seclusion room following the doctor’s prescription. This supports Adeniyi and Puzi (2021) when stating that non-pharmaceutical management of aggressive patients includes calming the patient, communication and de-escalations techniques. Also, the doctor should complete the *Mental Health Care Act* (MHCA) form 48 when secluding the patient. In pharmacological management, the patient should be sedated by treatment following a prescription by the doctor and should be done to calm the patient within a maximum of 2 h (Adeniyi & Puzi 2021).

The study participants reported the experience of verbal aggression by patients. In verbal aggression, according to Fortinash and Worret (2014), the patient makes a loud noise, shouts angrily, curses viciously, uses foul language, makes moderate threats to others or themselves, makes clear threats of violence towards other people or themselves and yells mild personal insults. The verbal aggression that nurses experienced in this study is well recorded in several studies of a similar nature (Hahn 2020; Fritz et al. 2020; Maluleke & Van Wyk 2017; Michael 2021; Stephens & Zile 2017; Weltens et al. 2021). Other forms of verbal aggression include verbal harassment with sexual content (Stevenson et al. 2015), harassment using sexual language and innuendos, and sexually inappropriate comments (Timori et al. 2019); participants in this study did not mention written threats of assault (Niu et al. 2019). Similarly, Mento et al. (2020) found that nurses become victims of aggression by patients and their relatives in the form of verbal abuse, physical assault, psychological violence and sexual abuse.

### Strengths and limitations

The current study was limited to determining the experiences of professional nurses working in psychiatric wards regarding the types of patient aggression. Other professionals might have different experiences. The recruitment and sampling strategy provided the sample with 2–25 years of working in a psychiatric ward, giving data characterised by a thorough experience of patients’ aggression. The study was conducted in a psychiatric ward; therefore, the results are not necessarily generalisable to other wards.

## Conclusion

The findings of the study revealed that nurses experience aggression of the patients which are physical, damage to property and verbal. These experiences were positive, indicating the existence of physical, damage to property and verbal types of aggression at the selected psychiatric hospitals. It is recommended that a qualitative study be conducted regarding the kind of support nurses need to manage aggressive patients.
